# The antitumor effect of diisopropylamine dichloroacetate on non-small cell lung cancer and its influence on the tumor immune microenvironment

**DOI:** 10.3389/fonc.2024.1447828

**Published:** 2024-08-29

**Authors:** Min Wei, Xiaoyan Shen, Ye Liu, Xiaotong Chen, Shu Su, Xin Lv, Xiaoping Qian, Lixia Yu, Lifeng Wang

**Affiliations:** ^1^ Department of Oncology, Nanjing Drum Tower Hospital, Nanjing Drum Tower Hospital Clinical College, Nanjing University of Chinese Medicine, Nanjing, China; ^2^ Department of Microbiology, Immunology and Pathology, Changzhou Hygiene Vocational Technology College, Changzhou, China; ^3^ The Comprehensive Cancer Centre of Nanjing Drum Tower Hospital, Affiliated Hospital of Medical School, Nanjing University and Clinical Cancer Institute of Nanjing University, Nanjing, China

**Keywords:** DADA, non-small cell lung cancer, pemetrexed, radiotherapy sensitization, tumor microenvironment

## Abstract

**Objective:**

To evaluate the antitumor effects of diisopropylamine dichloroacetate (DADA) alone or in combination with chemotherapy/radiotherapy/immunotherapy in NSCLC and explore the underlying mechanisms involved.

**Methods:**

MTT, UV spectrophotometry, flow cytometry, fluorescence microscopy, and clonogenic survival assays were used. In LLC mouse models, the antitumor effects of radiotherapy, DADA, and the anti-PD-1 antibody alone or in combination were evaluated, and the T cell numbers were evaluated in different groups.

**Results:**

DADA significantly inhibited lactate production and promoted apoptosis in NSCLC *in vitro*. Compared with pemetrexed or DADA alone, the combination of DADA with pemetrexed significantly inhibited proliferation and promoted apoptosis (p<0.05). This may be related to the decrease in the mitochondrial membrane potential in the combined group. Moreover, compared with radiotherapy alone, the combination of DADA with radiotherapy induced remarkable DNA damage. *In vivo*, the combination of radiotherapy, an anti-PD-1 antibody and DADA resulted in superior tumor inhibition than the combination of radiotherapy and anti-PD-1 antibody did (p < 0.05). The underlying mechanism may be partially related to the increased number of CD3+ T cells in the triplet combination group (p < 0.05).

**Discussion:**

Our results showed that DADA has strong antitumor effects on NSCLC, either alone or in combination with chemotherapy or radiotherapy. Interestingly, the combination of radiotherapy, anti-PD-1 antibody and DADA had a more pronounced tumor-suppressing effect, which may be related to DADA-induced T-cell generation by reducing local lactic acid production in the tumor microenvironment. This study lays the foundation for further exploration of DADA in lung cancer, especially in the era of immunotherapy, on the basis of its possible immunomodulatory effects.

## 1 Introduction

Globally, tumors caused by lung cancer are currently among the malignant tumors with the highest morbidity and mortality rates (1), and NSCLC accounts for more than 80% of all lung cancers (2). As most lung cancers have no obvious symptoms during the early stage of development, many patients have already lost the chance to undergo surgery when the tumors are found and need comprehensive antitumor treatment, mainly based on chemoradiotherapy. Although the application of new-generation cytotoxic drugs such as paclitaxel and pemetrexed has improved the prognosis of NSCLC patients to some extent, the effectiveness of chemotherapeutic drugs has reached a bottleneck and has been difficult to improve further for a long time. In recent years, with the application of targeted therapy in lung cancer, the treatment of advanced lung cancer has undergone rapid changes. The discovery of driver mutations and the application of corresponding targeted drugs have opened the door to precision treatment of lung cancer, and the optimization of treatment modalities and the exploration of novel drugs such as antibody−drug couplings will broaden the range of choices for patients with driver gene-positive NSCLC (3). Since the introduction of EGFR-TKIs, an increasing number of targeted drugs, such as ALK, ROS1, and RET, have improved the prognosis of patients, with remarkable efficacy. However, the high cost of targeted therapies in the clinical treatment of lung cancer and the inevitable primary or secondary resistance to almost all molecularly targeted drugs still limit their use (4). Furthermore, the application of immunotherapy in NSCLC has gradually progressed from advanced backline and first-line to perioperative treatment of early lung cancer, becoming a milestone in the field of lung cancer treatment. However, primary and secondary resistance to immunotherapy and the poor benefit of immunotherapy for patients with driver mutation-positive NSCLC are still therapeutic bottlenecks in the field. Previous studies have shown that the local immunosuppressive microenvironment of tumors is an important cause of immunotherapy resistance.

Malignant tumor cells have prominent metabolic differences from normal cells, which is mainly due to the “metabolic reprogramming” of tumor cells. In the classic example of the “Warburg effect”, tumor cells tend to supply energy via glycolysis even under oxygen-rich conditions due to mitochondrial respiratory dysfunction (5). Inhibition of key enzymes involved in glycolysis and reversal of the Warburg effect in tumor cells can be used as an antitumor therapeutic strategy. Dichloroacetate is a small-molecule metabolic modulator. In recent years, dichloroacetate, an inhibitor of pyruvate dehydrogenase kinase (PDK), has been shown to inhibit the inactivation of pyruvate dehydrogenase (PDH), the gating enzyme for the oxidative phosphorylation of glucose, which leads to inhibition of aerobic glycolysis, reduced lactic acid production in the tumor microenvironment, and increased mitochondrial membrane potential, which can inhibit the growth of tumor cells and achieve tumor suppression (6). In addition to its safety and tolerability, the antitumor effect of dichloroacetate has been confirmed for a variety of cancers, such as renal clear cell carcinoma, intestinal cancer, breast cancer, and endometrial cancer (7–10), and dichloroacetate has been demonstrated to exert a radiotherapy-sensitizing effect on cerebral gliomas and esophageal cancers (11, 12).

Diisopropylammonium dichloroacetate (DADA) is a derivative of dichloracetate, and this study aimed to explore the role of DADA in NSCLC. The antitumor effects and mechanisms of DADA in NSCLC were evaluated after its application alone or in combination with chemotherapy and radiotherapy. Based on the effect of DADA on tumor metabolism, we further hypothesized that DADA can modulate the tumor microenvironment via the inhibition of glycolysis and lactic acid production and further explored the *in vivo* antitumor effects and mechanisms of PD-1 inhibitors combined with DADA or local radiotherapy.

## 2 Material and methods

### 2.1 Cell lines

The human lung adenocarcinoma cell lines PC9, A549, HCC827, and LLC and the normal lung bronchial epithelial cell line HBE were purchased from American Type Culture Collection (ATCC) and stored in liquid nitrogen in the laboratory of the Oncology Center of Nanjing Gulou Hospital, Nanjing, China. The PC9, A549, HCC827, LLC, and HBE cell lines were grown as adherent monolayers, cultured in RPMI 1640 medium supplemented with 10% newborn calf serum, and cultured in a cell culture incubator at a constant temperature of 37°C with 5% CO_2_ and a humidified atmosphere.

### 2.2 Reagents

Pemetrexed was purchased from Eli Lilly Co., diisopropylammonium dichloroacetate (DADA) was purchased from Sanglid Biotechnology Co. (stored at 4°C, protected from light and dry, ready for use), the anti-γ-H2AX antibody was purchased from Abcam (Shanghai) Trading Co., the anti-mouse CD3, anti-mouse CD4, and anti-mouse CD8 antibodies were purchased from BD (Becton, Dickinson and Company, BD) Biotechnology, and the anti-PD-1 antibody was purchased from Suzhou Junmeng.

### 2.3 Cell proliferation assay

PC9, A549, HCC8227, and HBE cells were inoculated in 96-well plates (greinerbio-one, Germany) at 5×10^5^ cells/ml and allowed to adhere to the plate overnight (37°C); the next day, 200 µl of medium supplemented with different concentrations of DADA (0, 2, 4, 6, 8, 10, or 12 mmol/L) or pemetrexed (0, 0.0002, 0.002, 0.02, 0.2, 2, 20, or 200 µmol/L) was added to each well. MTT (Methylthiazolyldiphenyl-tetrazolium bromide (MTT) and dimethyl sulfoxide (DMSO) were added, the absorbance was measured, and the cell inhibition rate (IR) was calculated as follows: IR (%) = (control well OD value - experimental well OD value)/(control OD value - blank well OD value) × 100%. All experiments were performed in triplicate.

### 2.4 Cellular lactate production assay

PC9, A549, HCC8227, and HBE cells were inoculated in 6-well plates (greinerbio-one, Germany) at 4×10^5^ cells/ml and allowed to adhere overnight; the next day, the cells were treated with different concentrations of DADA (0, 2.5, 5, or 10 mmol/L) overnight; after 24 hours, trypsin digestion was performed for counting. The absorbance values of the samples were determined at 480 nm, and the lactate content was calculated as follows: lactate content of the culture medium (mmol/L) = (assay OD value - blank OD value)/(standard OD value - blank OD value) × standard sample concentration (3 mmol/L) × dilution of the sample before the test. A drug concentration–lactate production curve was then generated. All experiments were performed in triplicate.

### 2.5 Flow cytometry for detection of apoptosis and immunophenotyping

PC9 cells were inoculated in 6-well plates at 3-5×10^5^ cells/ml and cultured overnight. The next day, the cells were treated with different concentrations of DADA (0, 2.5, 5, 10, 15, or 20 mmol/L). After 48 hours of incubation, 3-5×10^6^ cells were collected, washed in PBS and resuspended in 1.5 ml of prechilled 1× binding buffer working solution. Aliquots were divided into three tubes: a blank control tube and two single-stained tubes. The single-stained tubes were supplemented with 3 µl of Annexin-FITC or 5 µl of PI, respectively, and the apoptosis rate was determined using a flow cytometer (BD, USA, BD FACSCanto™). All experiments were performed in triplicate.

The tumors of the mice in each group (LLC) were removed and ground into a cell suspension, and aliquots of 6×10^5^ cells/ml were placed in 1.5 ml Eppendorf tubes; 1 μL of fluorescent labeling antibody was added to each tube. The samples were incubated for 30 min protected from light, washed with NaCl and evaluated on the flow cytometer to obtain the data. Graphical data were gated with FlowJo software and analyzed to evaluate the expression of target proteins.

### 2.6 Evaluation of the mitochondrial membrane potential

Cells were seeded into plates and drugs were added as described above; the treated cells (1×10^5^ cells/ml) were placed in a 37°C incubator with 5% CO_2_ for 24 h and centrifuged (1000 r/min, 10 min), and 1 ml of JC-1 staining solution at a concentration of 1 mmol/L was added. The cells were incubated at 37°C for 30 min, and then the fluorescence intensity of the cells was observed by fluorescence microscopy.

### 2.7 Colony formation assays to verify the radiotherapy sensitization effect of DADA

PC9 cells were seeded in six 6-well plates (150 cells/well, 150 cells/well, 200 cells/well, 400 cells/well, 800 cells/well, and 1600 cells/well) and allowed to adhere overnight; the cells were then treated with 3 mmol/L DADA and incubated for 24 h. Next, the cells were irradiated with a total dose of 0 Gy, 1 Gy, 2 Gy, 4 Gy, 6 Gy, or 8 Gy; the medium was changed the day after radiotherapy, and the cells were incubated for 10-14 days. The resulting colonies were stained with 0.5% crystal violet and counted under a microscope, and ≥50 cells were counted as one colony under low magnification. The dose−survival fraction curves were fitted according to the single-click multitarget model [SF=1- (1-e^KD^) ^N^] using GraphPad Prism 9 software. All experiments were performed in triplicate.

### 2.8 Immunofluorescence

DNA double-strand breaks (DSBs) were detected by immunofluorescence staining of phosphorylated H2AX. Cells were seeded into 6-well plates, subjected to 8 Gy of radiation, incubated for 5 hours, fixed with 4% neutral formaldehyde for 30 minutes at room temperature and then incubated at 4°C overnight with the primary antibody. The next day, the γ-H2AX primary antibody was removed, and the cells were incubated overnight at 4°C with the secondary antibody. The next day, the secondary antibody was removed, and the cells were incubated for 2 hours. DAPI was used to stain the cell nuclei, and the cells were visualized by fluorescence microscopy.

### 2.9 Subcutaneous tumor graft model

Five-week-old male C57L/6 mice (20 ± 2 g) were purchased from Qinglongshan Animal Breeding Center, Jiangning District, Nanjing City, China, and 2×10^6^ LLC cells were injected subcutaneously into the left groin. The mice were randomly divided into the following five groups when the tumor volume reached 80-100 mm^3^: control group, single-dose 8 Gy radiotherapy, radiotherapy combined with DADA (50 mg/kg, perfused for 7 days), radiotherapy combined with anti-PD-1 antibody (50 mg/kg), radiotherapy combined with anti-PD-1 antibody and DADA. Tumor volume and body weight were recorded for further analysis.

### 2.10 Statistical analysis of the data

SPSS 21.0 statistical software was used for statistical analysis of the data. All measurement data are expressed as the mean ± standard deviation (mean ± s), and comparisons among multiple groups were performed by one-way ANOVA, with a two-sided p<0.05 indicating a statistically significant difference.

## 3 Results

### 3.1 DADA inhibits the proliferation of lung cancer cells

**Figure 1:**
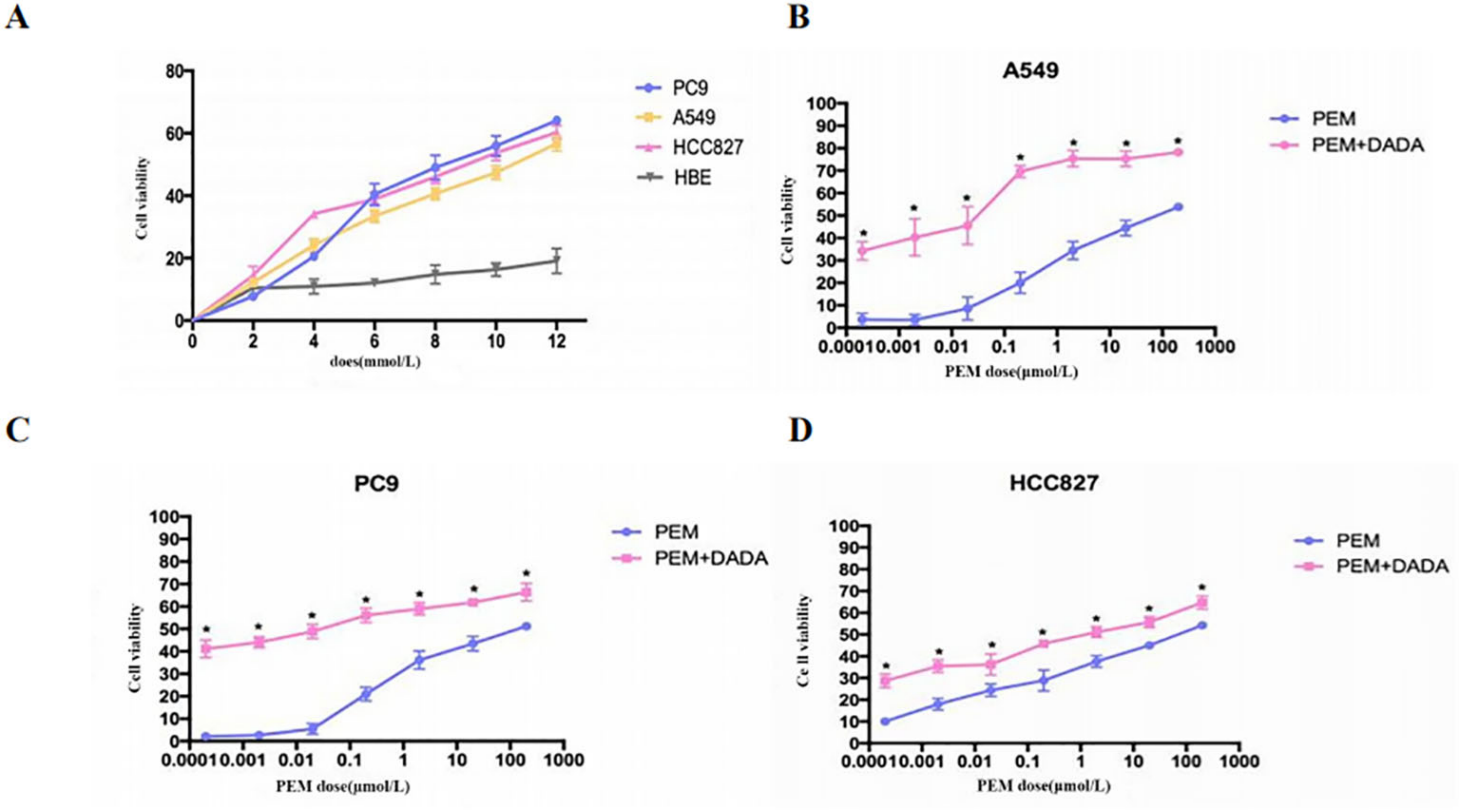
Cell proliferation determined by an MTT assay. Serial dilutions of DADA and pemetrexed solutions were added to PC9, A549, HCC827, and HBE cells, which were then incubated for 48 h. After incubation, cell viability was evaluated via the MTT assay. The viability of the untreated cells was set to 100% as control. **(A)** Rate of cell proliferation inhibition by different concentrations of DADA; **(B–D)** Rate of cell proliferation inhibition by pemetrexed alone or in combination with 5 mmol/L DADA. (* indicates that the rate of inhibition of cell proliferation in the combination group was greater than that in the pemetrexed monotherapy group at the same concentration of pemetrexed, and the difference was statistically significant; p<0.05).

Serially diluted DADA and pemetrexed solutions were added to PC9, A549, HCC827, and HBE cells, and cell viability was evaluated using an MTT assay after 48 hours of incubation. When the concentration of DADA was ≥4 mmol/L, the extent to which DADA inhibited proliferation was significantly greater for tumor cells than for normal HBE lung epithelial cells (p<0.05). As the concentration of DADA increased, the rate of inhibition of tumor cell proliferation increased, but there was no significant effect on the proliferation of normal HBE lung epithelial cells (**Figure 1A**). To further validate the synergistic effect of DADA combined with chemotherapeutic drugs, PC9, A549, and HCC827 cells were treated with different concentrations of pemetrexed combined with DADA, and the rate of proliferation inhibition for the cells treated with the drug combination was greater than that for cells treated with the single agent pemetrexed (p<0.05), as shown in **Figures 1B–D**.

### 3.2 Effect of DADA on lactate production in tumor cells

**Figure 2:**
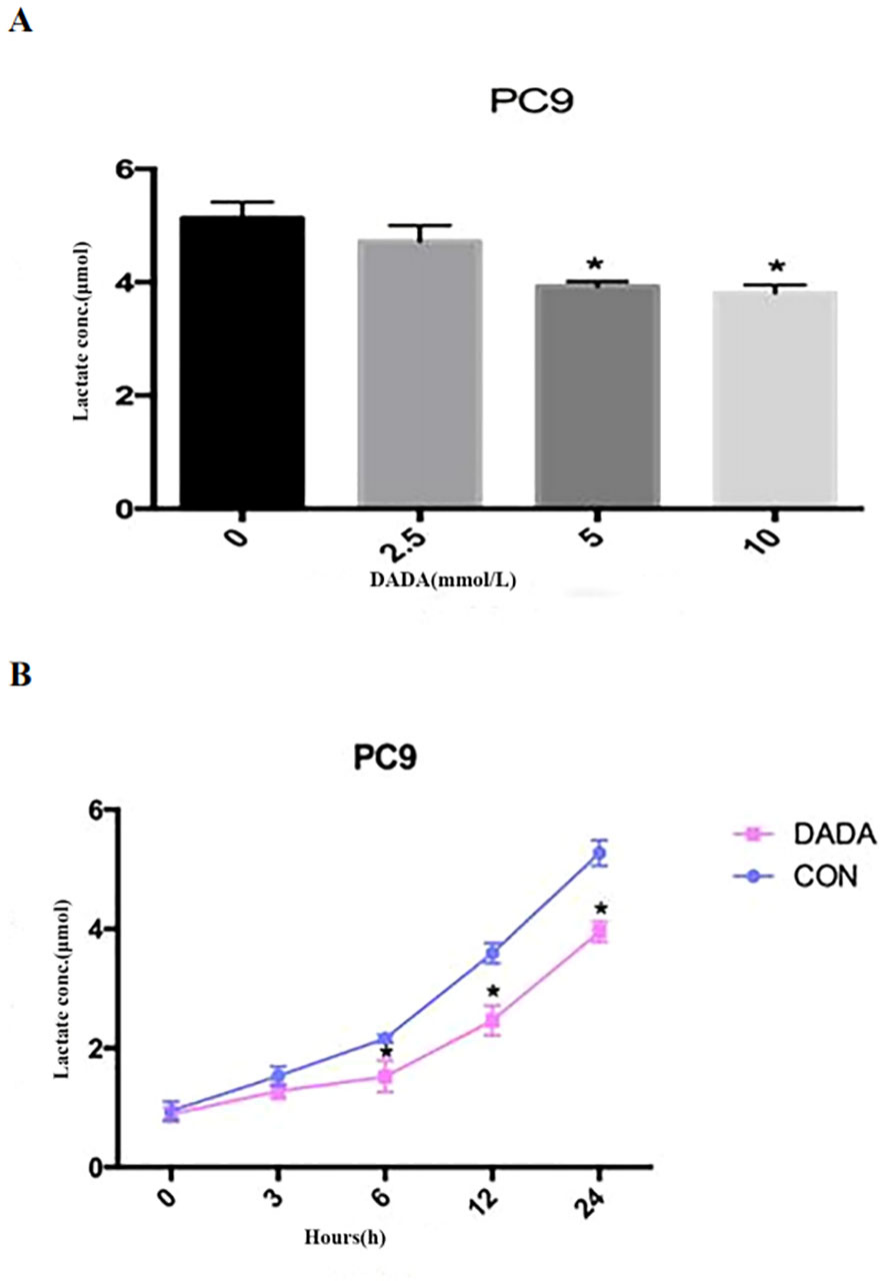
Inhibition of lactate secretion by DADA. **(A)** Lactate production per 10^5^ cells in culture medium after treatment with different concentrations of DADA for 24 hours; **(B)** Lactate production per 10^5^ cells in culture medium after treatment with 5 mmol/L DADA for different durations. (* indicates that the lactate content in the culture medium after treatment with DADA at this concentration was significantly lower than that in the culture medium of the untreated group; p<0.05).

PC9 cells were treated with serially diluted DADA for 24 h. As the concentration of DADA increased, the amount of lactate released into the culture medium per 10^5^ cells gradually decreased after 24 h. When the concentration of DADA was ≥5 mmol/L, the amount of lactate produced per 10^5^ PC9 cells was less than that in the untreated group (p<0.05) (**Figure 2A**). Further exploration of the effect of 5 mmol/L DADA on lactate production at different time points in PC9 cells revealed that when the duration of treatment was ≥6 hours, the amount of lactate released into the culture medium of the treated group was lower than that in the culture medium of the control group (**Figure 2B**).

### 3.3 Effect of DADA alone or in combination with pemetrexed on apoptosis

**Figure 3:**
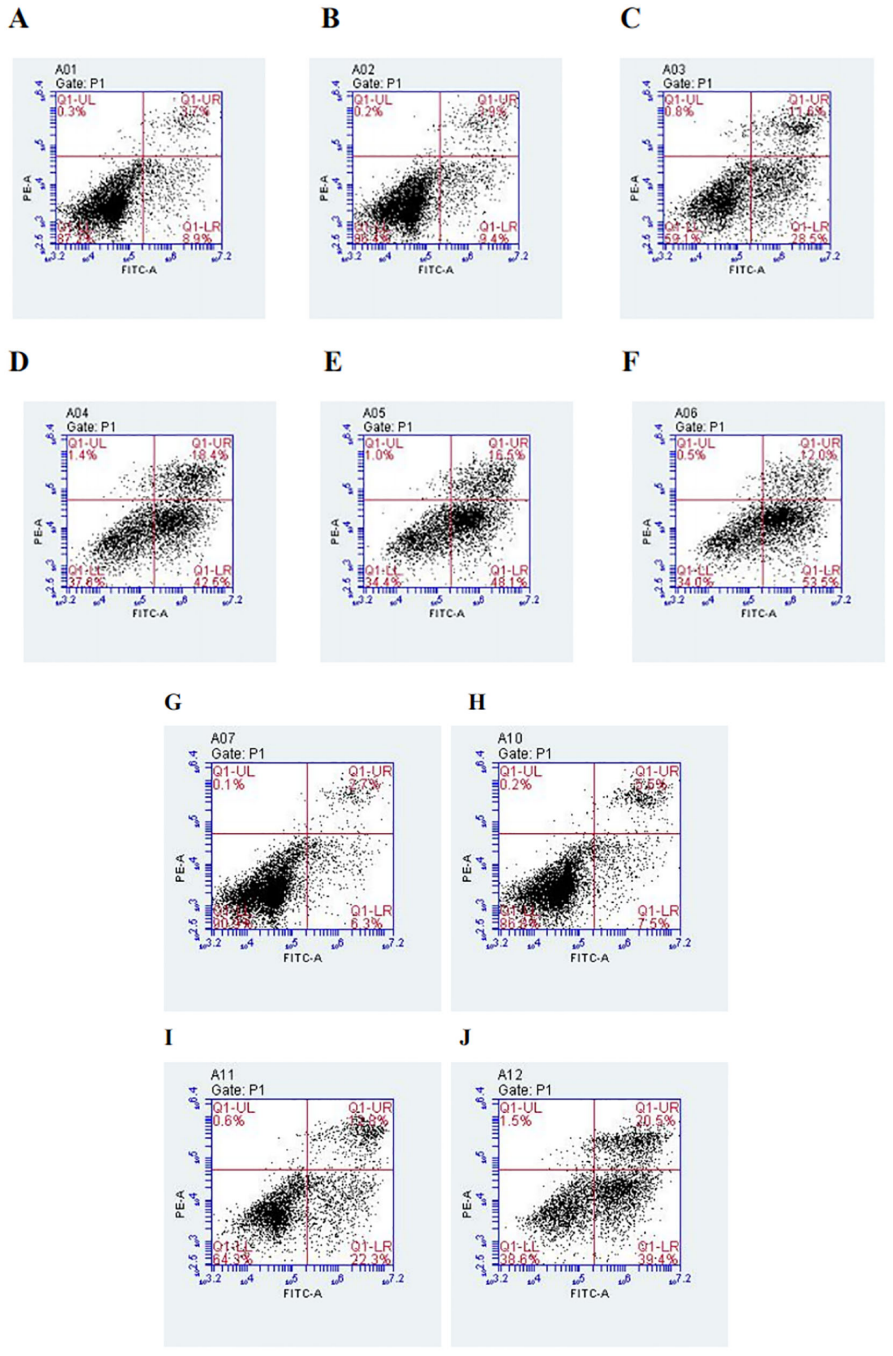
Effect of treatment with different concentrations of DADA on PC9 cell apoptosis and different treatments on PC9 cell apoptosis. **(A)**, Control group; **(B–F)**, Groups treated with 2.5, 5, 10, 15, and 20 mmol DADA. The higher the concentration of DADA, the more pronounced the pro-apoptotic effect.(Lower right quadrant: early apoptosis; upper right quadrant; late apoptosis). **(G)**, Control group; **(H)**, Pemetrexed single-agent treatment group; **(I)**, DADA single-agent treatment group; **(J)**, Pemetrexed + DADA treatment group. The apoptosis rate of the combined group was greater than that of the control group or the single-drug groups. (Lower right quadrant: early apoptosis; upper right quadrant: late apoptosis).

After PC9 cells were treated with different concentrations of DADA alone (at a concentration of ≥5 mmol/L) for 48 hours, the percentage of apoptotic cells in the DADA-treated group was significantly greater than that in the control group (p<0.05). In addition, the greater the concentration of the drug was, the greater the degree to which apoptosis was induced by DADA (**Figures 3A–F**). The results showed that DADA promoted apoptosis to some degree; therefore, we further hypothesized that 5 mmol/L DADA combined with 0.02 µmol/L pemetrexed would promote apoptosis more strongly than DADA alone, and the results showed that apoptosis was more frequent (p<0.05) in the DADA combined with pemetrexed group than in the DADA alone group (**Figures 3G–J**).

### 3.4 Effect of DADA in combination with chemotherapy on the mitochondrial membrane potential in tumor cells

**Figure 4:**
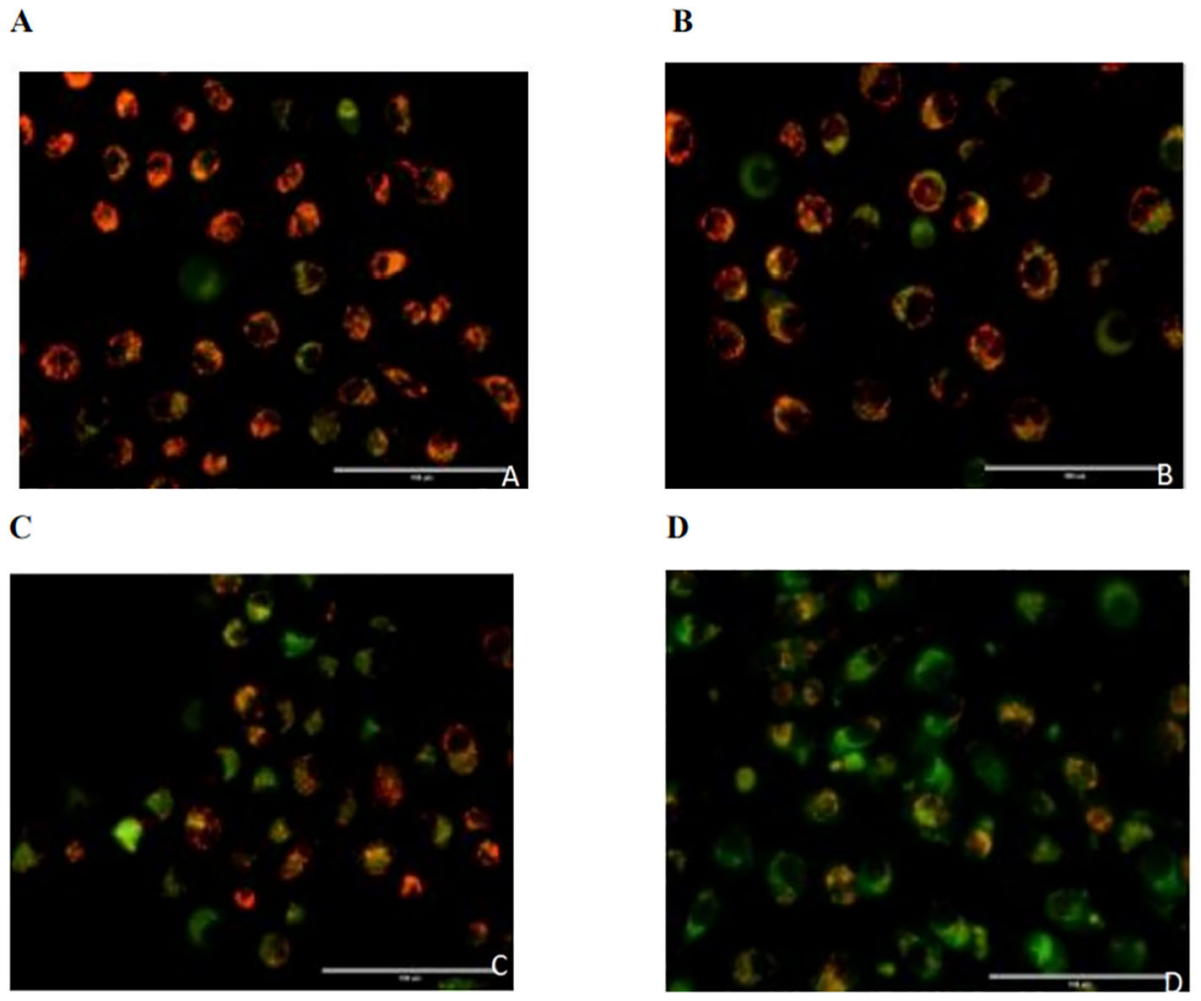
Effects of different treatments on the mitochondrial membrane potential of PC9 cells. **(A)**, Control group; **(B)**, Pemetrexed monotherapy group; **(C)**, DADA monotherapy group; **(D)**, Pemetrexed +DADA treatment group: When the mitochondrial membrane potential decreased, the red fluorescence shifted to green fluorescence, and the mitochondrial membrane potential of the combined group was lower than that of the control group and the single agent group(p<0.05).

The red/green fluorescence ratios measured for the control group, 0.02 µmol/L pemetrexed alone group, 5 mmol/L DADA alone group, and pemetrexed combined with DADA group after 48 hours of treatment are presented in **Table 1**. Red fluorescence shifts to green fluorescence when the mitochondrial membrane potential decreases. Fluorescence microscopy revealed that the percentage of red JC-1-positive PC9 cells in the combined drug treatment group was significantly lower than that in the single drug group(p<0.05), which indicated that the mitochondrial membrane potential in the combined treatment group was lower than that in the pemetrexed and DADA single drug groups (**Figures 4A–D**).

**Table 1:** Intensities and ratios of red and green fluorescence in each treatment group.

process	red fluorescent intensity	green fluorescence intensity Green	red/green fluorescence ratio
control	36.047	10.367	3.477
pemetrexed	13.089	6.695	1.955
DADA^a^	9.987	10.976	0.910
pemetrexed +DADA	9.972	23.093	0.432

^a^diisopropylamine dichloroacetate.

### 3.5 DADA-mediated sensitization of the PC9 cell line to radiotherapy

**Figure 5:**
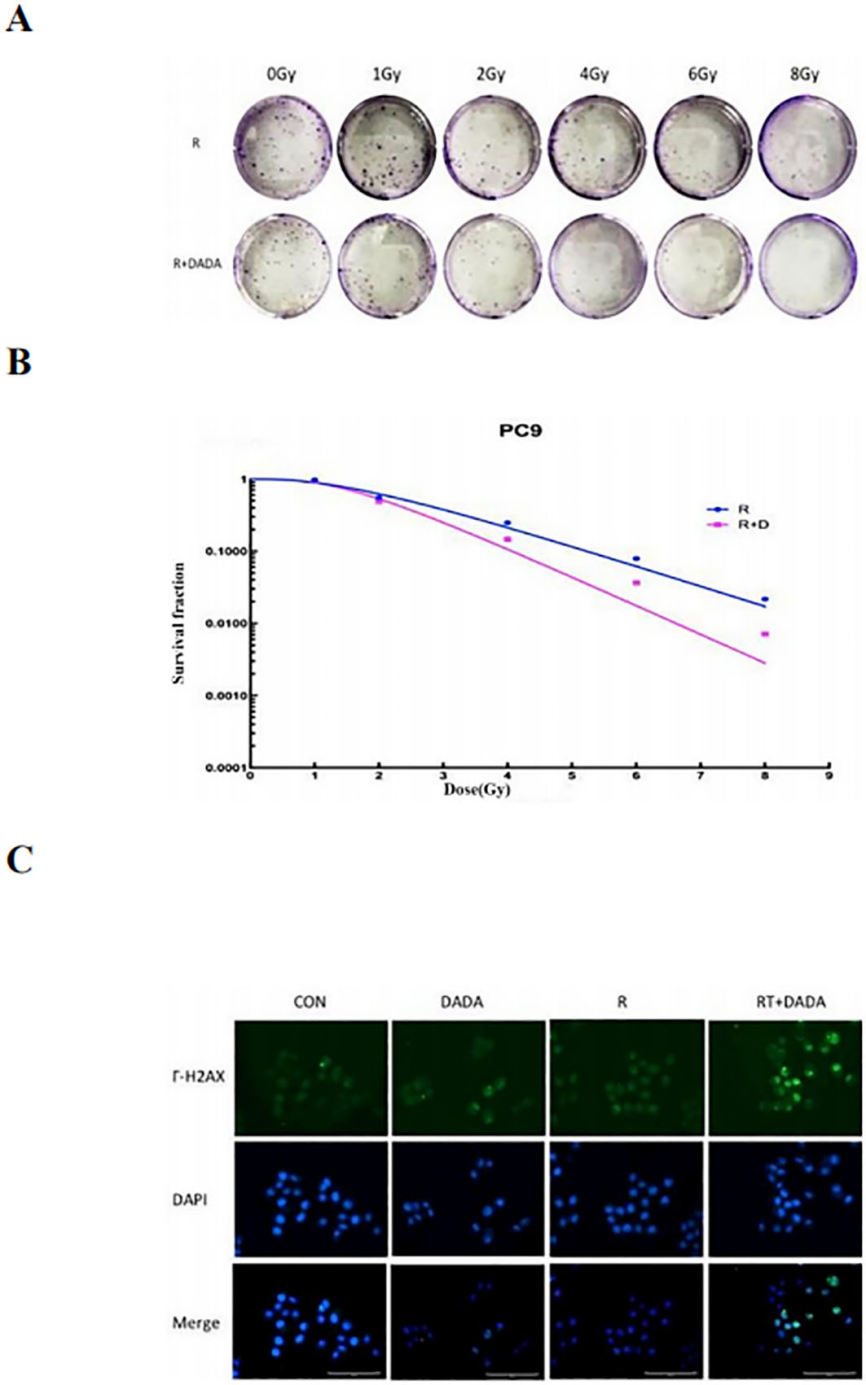
Radiotherapy-sensitizing effect of DADA. **(A)** Images of colony formation in each group after treatment with radiotherapy alone or in combination with DADA; **(B)** Radiation dose-survival fraction fitting curves. **(C)** γ-H2AX expression in the control group, DADA alone group, radiotherapy group, and radiotherapy combined with DADA group (blue represents the nucleus, green represents the γ-H2AX protein).

After a 2 Gy dose of radiotherapy, the survival fraction (SF2) was lower in the DADA combined with radiotherapy group than in the radiotherapy alone group, and the sensitization enhancement ratio (SER) was 1.42 (**Table 2**). These data indicated that radiotherapy combined with DADA increased cell death in PC9 cells compared to that observed after radiotherapy alone (**Figures 5A, B**). To further explore the radiosensitization effect of DADA on PC9 cells, we detected γ-H2AX, a marker of DNA double-strand breaks, whose expression is positively correlated with the amount of DNA damage after radiotherapy. After 48 h, fluorescence microscopy revealed that the fluorescence intensity of γ-H2AX in the DADA-treated PC9 cells was greater than that in the DADA-alone and radiotherapy-alone groups (**Figure 5C**), which indicated that the combined treatment caused more DNA damage than did DADA alone or radiotherapy alone.

**Table 2:** Ability of DADA to sensitize PC9 cells to radiotherapy *in vitro*.

process	D0^a^	Dq^b^	SF2^c^	SER^d^
RT^e^	1.55	1.72	0.62	
RT+DADA	1.09	1.60	0.53	1.42

^a^D0: mean lethal dose, ^b^Dq: quasithreshold dose, ^c^SF2: survival fraction after 2 Gy, ^d^SER: sensitization enhancement ratio, ^e^radiotherapy.

### 3.6 Radiosensitization effect of DADA *in vivo* and ability to modulate the tumor immune microenvironment

**Figure 6:**
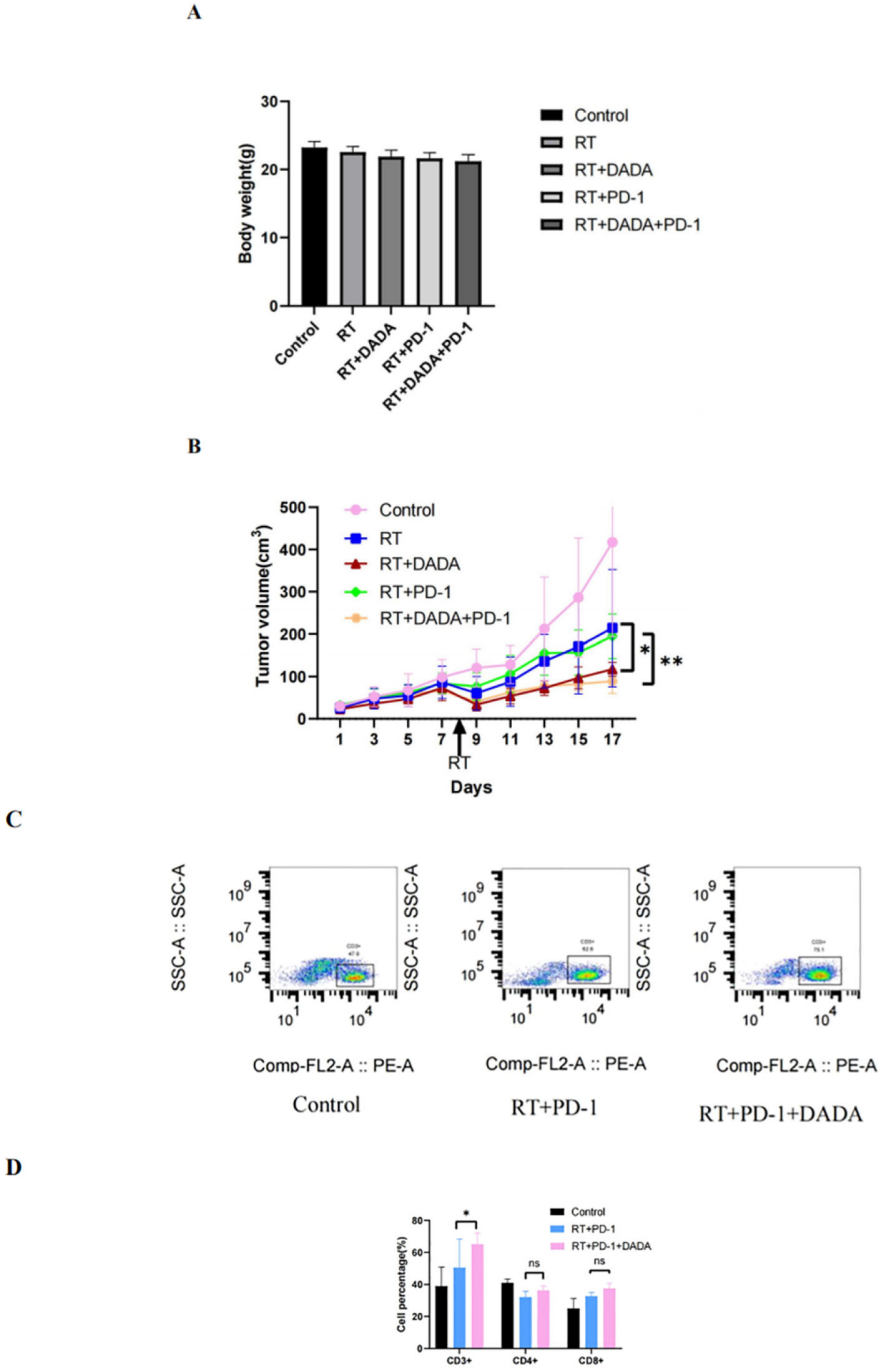
Effects of different combinations of DADA, RT, and anti-PD-1 antibodies *in vivo*. **(A)** Changes in body weight in the control group, radiotherapy group, radiotherapy combined with DADA group, radiotherapy combined with anti-PD-1 antibody group, and radiotherapy/DADA/anti-PD-1 antibody triple combination group at 10 days after radiotherapy. **(B)** Tumor volume over time in the control group, radiotherapy group, radiotherapy combined with DADA group, radiotherapy combined with anti-PD-1 antibody group, and radiotherapy/DADA/anti-PD-1 antibody triple combination group at 10 days after radiotherapy. [*: The tumor volume in the radiotherapy combined with DADA group was smaller than that in the radiotherapy alone group (p<0.05);**: the tumor volume in the radiotherapy/DADA/anti-PD-1 antibody triple combination group was smaller than that in the radiotherapy combined with anti-PD-1 antibody group (p<0.05)]. **(C)** Percentages of CD3+ T cells in the tumor tissues of mice in the control group, radiotherapy combined with anti-PD-1 antibody group, and radiotherapy+DADA+anti-PD-1 antibody triple combination group. **(D)** Percentages of CD3+CD4+CD8+ T cells in the tumor tissues of mice in the control group, radiotherapy combined with anti-PD-1 antibody group, and radiotherapy+DADA+anti-PD-1 antibody triple combination group (*: CD3+ T-cell abundance in the radiotherapy+DADA+anti-PD-1 antibody triple combination group was greater than that in the radiotherapy combined group; p<0.05). ns: no statistical difference between RT+PD-1 group versus RT+PD-1 +DADA group in terms of CD4+ T cells and CD8+ T cells.

We established a subcutaneous tumor transplantation model in C57L/6 mice using LLC cells. The weight and tumor volume of the mice were recorded during the observation period (**Figures 6A, B**). The results revealed that there was no significant difference in the weight of the mice between groups. However, the tumor volume in the radiotherapy combined with DADA group was smaller than that in the radiotherapy alone and radiotherapy combined with PD-1 monoclonal antibody groups (p<0.05); these results indicated that DADA could sensitize the mice to radiotherapy *in vivo*. Previous studies have shown that radiotherapy and immunotherapy can exert synergistic antitumor effects (13), so we further explored whether the addition of DADA could increase treatment efficacy or whether the local immune microenvironment of the tumor could impact the therapeutic effect of radiotherapy combined with anti-PD-1 antibody. The results showed that the tumor volume in the combined DADA+radiotherapy+anti-PD-1 antibody group was smaller than that in the radiotherapy combined with anti-PD-1 antibody group (p<0.05). Furthermore, cell suspensions of tumor tissues were generated and analyzed in a flow cytometric phenotypic assay. CD3+ T-cell expression in the DADA+radiotherapy+anti-PD-1 antibody group was greater than that in the radiotherapy combined with anti-PD-1 antibody group (p<0.05), and there were no significant differences in CD4+ or CD8+ T-cell numbers (**Figures 6C, D**).

## 4 Discussion

Lung cancer is a type of malignant tumor with one of the highest morbidity and mortality rates (1). Non-small cell lung cancer (NSCLC) patients account for approximately 85% of lung cancer patients, and the vast majority of these patients are diagnosed at advanced stages and require drug-based treatment. In recent years, the emergence of new treatment methods and drugs has led to a significant decrease in lung cancer mortality, but the five-year survival rate of NSCLC patients is still less than 20%, and new therapeutic approaches need to be explored to improve the prognosis of patients. The energy metabolism of tumor cells can regulate cell proliferation, growth, migration, invasion, and other important biological activities, thus playing an important role in tumor development and progression. Therefore, targeting tumor metabolism is very important for tumor therapy. In recent years, an increasing number of drugs targeting the metabolism of tumor cells or immune cells have been developed (14, 15) and have broad development prospects.

Dichloroacetate has been used as a small-molecule metabolic modulator in the treatment of human lactic acidosis and hereditary mitochondrial diseases for almost 40 years (16). In recent years, researchers have shown that dichloroacetate, a PDK inhibitor, inhibits the inactivation of PDH, the gating enzyme for the oxidative phosphorylation of glucose, and as a result, inhibits aerobic glycolysis in tumor cells, reduces lactate production in the tumor microenvironment, and decreases the mitochondrial membrane potential, which in turn inhibits the growth of tumor cells and achieves tumor-suppressive effects (6). In addition to its safety and tolerability, the antitumor effect of dichloroacetate has been confirmed in a variety of cancers, such as renal clear cell carcinoma, intestinal cancer, breast cancer, and endometrial cancer (7–10), and dichloroacetate has been shown to have radiotherapy-sensitizing effects on glioma and esophageal cancer (11, 12).

DADA has the active ingredient of dichloroacetate. This study is the first to explore the effect of DADA on tumors and the potential chemotherapy- and radiotherapy-sensitizing effects of DADA in the context of lung cancer and to further explore the ability of DADA to modulate the tumor immune microenvironment based on its metabolic effects. *In vitro* studies revealed that DADA had significant inhibitory effects on cell proliferation, reversed the changes in energy metabolism observed in tumor cells, and induced tumor cell death in lung adenocarcinoma cells. DADA had synergistic antitumor effects when combined with pemetrexed, as indicated by a greater percentage of apoptotic cells in the DADA group than in the pemetrexed group. Mechanistic analysis suggested that the combination of DADA and pemetrexed significantly inhibited aerobic glycolysis, reduced lactate production in the tumor microenvironment, and decreased the mitochondrial membrane potential, thereby limiting tumor cell growth. Previous reports have shown that DADA inhibits cellular proliferation and reduces lactate secretion in other tumor types, thereby inhibiting tumor growth (10, 17), which was consistent with the results of the present study on lung cancer, providing evidence for the potential use of DADA in cancer therapy.

Radiotherapy is an important treatment modality for NSCLC, but the resistance of tumor cells to radiation and the damage to normal cells caused by high-dose radiation are the two major obstacles limiting tumor radiotherapy. Therefore, exploring radiotherapy-sensitizing drugs and enhancing the sensitivity of tumor cells to radiotherapy is an important approach for improving treatment efficacy. Our study revealed that DADA combined with radiotherapy induced the production of γ-H2AX compared with radiotherapy alone, suggesting that DADA could enhance the DNA damage caused by radiotherapy and thus enhance the efficacy of radiotherapy. Further *in vivo* experiments showed that radiotherapy combined with DADA inhibited tumor growth better than radiotherapy alone or radiotherapy combined with an anti-PD-1 antibody. These findings suggest that inducing the transition of tumor cells from glycolysis to mitochondrial oxidation may be an attractive approach to increase the sensitivity of cancer cells to radiotherapy in the future.

Immunotherapy has revolutionized the field of NSCLC treatment and has gradually become a routine therapy. Despite significant clinical improvements, a considerable number of patients do not benefit from ICI therapy due to primary or secondary drug resistance. With further research on tumor immunity, it has gradually become clear that the complexity and diversity of the tumor microenvironment (TME) are key factors affecting the efficacy of immunotherapy. Recent findings have shown that dichloroacetate can target cancer cells and inhibit PDK, shifting tumor metabolism from glycolysis to oxidative phosphorylation, thereby reducing lactate production in tumor cells and promoting T-cell activation and inhibiting tumor growth (18, 19). Therefore, based on *in vitro* experiments demonstrating the effects of DADA on the metabolism of tumor cells and the mitochondrial membrane potential, we hypothesized that DADA could promote antitumor responses through its effects on the local immune microenvironment of tumors during anti-PD-1 therapy. Our *in vivo* results showed that the inhibitory effect of the triple combination of DADA+radiotherapy+anti-PD-1 antibody on tumor growth was significantly greater than that of radiotherapy combined with anti-PD-1 antibody; further analysis revealed that in the DADA+radiotherapy+anti-PD-1 antibody group, the number of CD3+ T cells in tumor tissues was significantly greater than that in the radiotherapy combined with anti-PD-1 antibody group. We speculate that DADA can improve the tumor microenvironment by decreasing the lactate concentration in tumor tissues and decreasing the mitochondrial membrane potential (20), thus promoting T-cell proliferation and activation and restoring the cellular immune response to some extent. Further T-cell subpopulation analysis, including an analysis of CD4+ and CD8+ T-cell expression, revealed no significant differences, which may have been due to the insufficient sample size. In subsequent experiments, we will use a larger sample size for multidimensional analysis of the effect of DADA on the tumor microenvironment.

Although progress has been made in the fields of molecular targeting and immunotherapy in the treatment of NSCLC in recent years, drug resistance to related drugs is inevitable, which seriously affects patient prognosis and survival. Previous studies have shown that the overall immunotherapeutic efficacy of driver mutation-positive NSCLC agents, such as EGFR, is unsatisfactory, possibly because of the local immune-negative microenvironment of the tumor after EGFR TKI resistance. In addition, for immunotherapy, the immune microenvironment is also an important reason for primary or secondary resistance to immunotherapeutic drugs, and modification of the local immune microenvironment of the tumor is important for overcoming drug resistance. In the present study, we demonstrated for the first time through *in vitro* and *in vivo* experiments that DADA inhibits cell proliferation, promotes tumor cell apoptosis, reduces lactic acid production, and enhances the therapeutic effect of radiotherapy and chemotherapy in the context of lung cancer. The sensitizing effect of DADA on radiotherapy and chemotherapy was confirmed. On the basis of the results of previous studies, DADA further affects tumor metabolism through its effects on lactate production and cellular mitochondrial membrane potential. We further hypothesized that DADA has a regulatory effect on the local immunonegative microenvironment of tumors. Preliminarily, *in vivo* findings also revealed that the addition of DADA could further enhance antitumor effects by increasing the accumulation of T cells in tumor tissues after the combined application of radiotherapy and anti-PD-1 antibody, suggesting that the immune-modulating effect of DADA on the tumor microenvironment has broad application prospects for future immunotherapy-based treatment strategies. Based on the results of the existing studies, further exploration of the effects of the application of DADA in combination with molecularly targeted drugs or immunotherapeutic agents on the local microenvironment of the tumor and the antitumor effects at different stages of the disease is worthwhile.

## Data Availability

The original contributions presented in the study are included in the article/supplementary material. Further inquiries can be directed to the corresponding author.
